# Innovative Insights into Fatigue Damage Evolution of Shield Tunnel Segments by Introducing Stable Damage Limit and Memory Effect

**DOI:** 10.3390/ma18040740

**Published:** 2025-02-07

**Authors:** Yang Zou, Yichao Ye, Qianlong Tang

**Affiliations:** 1 School of Civil Engineering, Central South University, Changsha 410075, China; 184801013@csu.edu.cn; 2 LETS GROUP Co., Ltd., Xiamen 361004, China; tunnelye@163.com

**Keywords:** fatigue initial damage, fatigue damage characteristics, damage evolution, nonlinear strain development, structural concrete design

## Abstract

In this study, an experimental equipment is designed to simulate the mechanical boundary conditions of shield tunnels and test samples that exhibit varying degrees of initial damage fabricated through low-energy impact experiments. The fatigue damage characteristics of the initially damaged segments are examined under multistage cyclic loading. A comprehensive analysis of dynamic strain, mean strain, and strain amplitude time–history curves is conducted to derive damage evolution curves based on the degradation of elastic modulus. The average damage evolution rate is evaluated from these curves. The findings reveal that both initial damage and load level significantly influence segment performance evolution, potentially increasing the damage evolution rate by tenfold or more. Importantly, the results highlight a “memory effect” in damage evolution and identify a critical threshold that distinguishes between “stable” and “accelerating” damage evolution stages. Exceeding this threshold results in a substantial acceleration of the damage evolution rate, ultimately leading to failure. This research introduces the innovative concept of a stable damage limit and underscores the crucial role of initial damage in the nonlinear strain development of segmented concrete, providing valuable insights for improving fatigue design strategies in structural concrete elements.

## 1. Introduction

As urban underground spaces continue to develop, the operational environment for shield tunnels is increasingly complex [1,2]. Specifically, the repetitive train loads can lead to cumulative deformation of tunnel segments, resulting in damage, cracking, water leakage, and concrete corrosion. These issues significantly impact the structural integrity and safe operation of subway systems [3]. Addressing these challenges is crucial for the design of concrete fatigue resistance in shield tunnel segments.

Domestic and foreign scholars have carried out a series of studies on the damage and fatigue of concrete in shield tunnels. Regarding the factors influencing concrete fatigue, Li et al. [4] examined the effects of high-speed train aerodynamics on cumulative fatigue damage, while Sun et al. [5] investigated how free water impacts concrete fatigue properties. Yan et al. [6] used numerical methods to explore the dynamic response and cumulative damage evolution in overlapping shield tunnels. Deng et al. [7] focused on fatigue fracture characteristics in concrete with cold joint defects, and Huang et al. [8] analyzed the performance of prestressed concrete beams under combined corrosion and fatigue conditions. Wang et al. [9] demonstrated that corrosion significantly affects the fatigue performance of reinforced concrete beams, reducing their ultimate strength and ductility through flexural tests. Wei et al. [10] researched the fatigue damage in perforated shear joints and geometric configurations, and Miarka et al. [11] studied the high-cycle fatigue life of notched concrete specimens. Concerning loading modes, Zare et al. [12] highlighted the significant impact of strain amplitude on cyclic characteristics in loading tests. Liu and Zhou [13] explored fatigue strain development in reinforced concrete beams under constant amplitude bending loads, while Wang and Song [14] proposed a fatigue failure criterion for concrete under constant lateral stress. Keerthana and Kishen [15] developed a fatigue model for plain concrete under varying loads, considering stress ratio and overload effects on crack growth. Lantsoght et al. [16] introduced a new compressive fatigue test analysis method for repeated high-cycle loads. Miarka et al. [11] also provided enhanced fatigue life predictions through high-cycle fatigue tests under three-point bending, and Skarzynski et al. [17] investigated fatigue properties of plain concrete under uniaxial compression, reporting a 30% increase in total crack volume under cyclic loading in comparison with normal loading. In terms of testing methods and analysis, Karr et al. [18] indicate that for undamaged concrete specimens, no fatigue limit exists below 1 billion cycles at a maximum compressive stress of 0.44 f(c). Fan et al. [19] used computed tomography to calibrate fatigue damage. Utilizing an improved concrete constitutive model under uniaxial cyclic loading, Yan et al. [20] demonstrates that the failure of tunnel structures is primarily governed by tensile damage, with the maximum principal stress at the tunnel crown being at least five times greater than that at the crown. Gao et al. [21] proposed a model simulating strain development in concrete and steel bars under fatigue loads, while Zanuy et al. [22] developed a model to analyze reinforced concrete columns and beams’ response under fatigue. Chen et al. [23] created a fatigue life prediction method for concrete structures under mixed loads, and Guo et al. [24] introduced a fatigue model within the Cosserat cyclic dynamic framework to study concrete crack propagation. Doroudi et al. [25] established a model for damage evolution under cyclic fatigue loads, and Ghatee et al. [26] developed a nonlinear damage formula with calibration capabilities for tensile and compression states. Mazars et al. [27] proposed a model integrating elasticity and damage coupling.

Furthermore, various construction-related issues often lead to unavoidable initial damage in the segment structures of tunnels [28,29,30]. Such initial damage can progressively worsen under cyclic loading once the tunnel is operational, posing significant risks to its safe functionality [31]. Tian et al. [32,33] developed a three-dimensional numerical model of shield tunnel linings with initial defects, analyzing the fracture characteristics and failure mechanisms of lining segments to accurately estimate their fatigue life. Wang et al. [33] investigated how cracks of varying lengths, numbers, and locations affect damage evolution in tunnel segments through model testing.

The aforementioned studies primarily target the material level and are constrained by cyclic loading parameters and boundary conditions. Due to the complex damage mechanisms involved in segmented concrete under multi-stage cyclic loads, numerical analyses often do not yield accurate and reliable results. Experimental approaches that include applying mechanical boundaries to segments can more effectively illuminate the mechanical behavior related to segment concrete fatigue damage. Unfortunately, there is a paucity of research in this area. To better investigate the damage evolution mechanism of initially damaged segmental concrete under vehicle-induced cyclic loading and to predict its fatigue life, this paper proposes designing a test device capable of simulating the mechanical boundaries of shield tunnel structures. Utilizing an MTS hydraulic servo loading system, we conduct fatigue failure tests to examine the deformation behavior and damage characteristics of shield tunnel segment concrete under multi-stage cyclic loads. This study presents relevant test methods and evolution curves to elucidate the damage and failure characteristics and underlying mechanisms.

## 2. Test Program

### 2.1. Test Equipment

**Figure 1:**
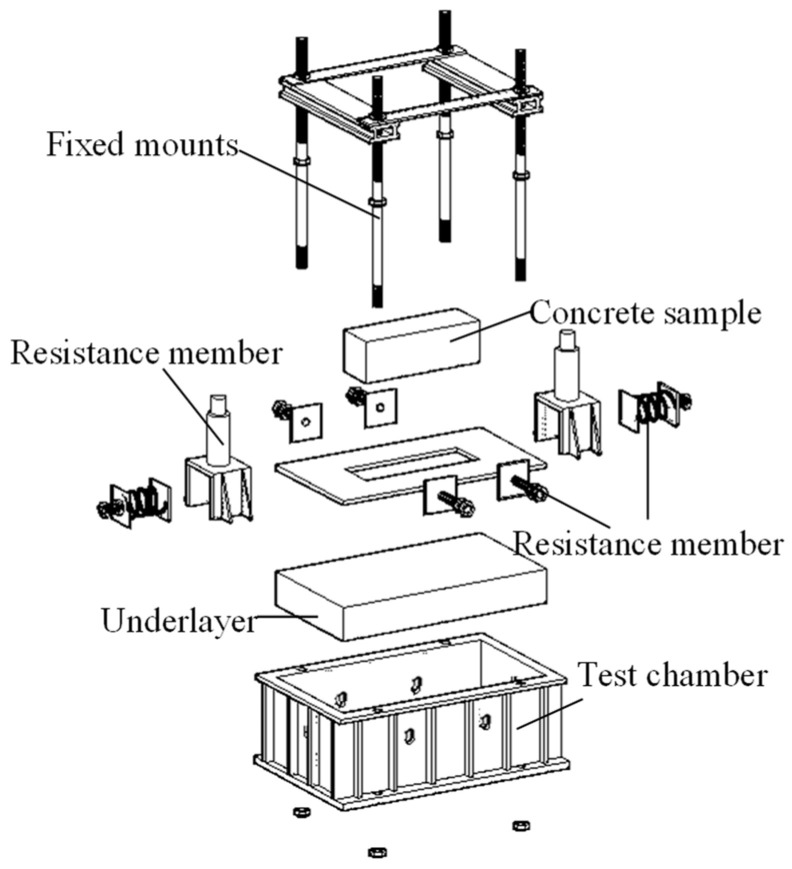
Component units of test equipment.

**Figure 2:**
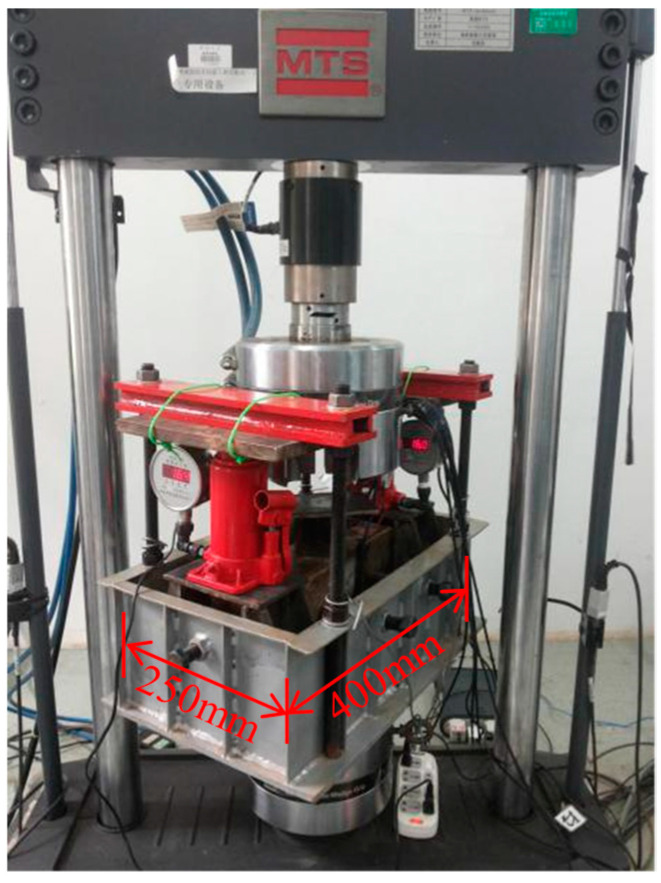
Cyclic loading test.

**Figure 3:**
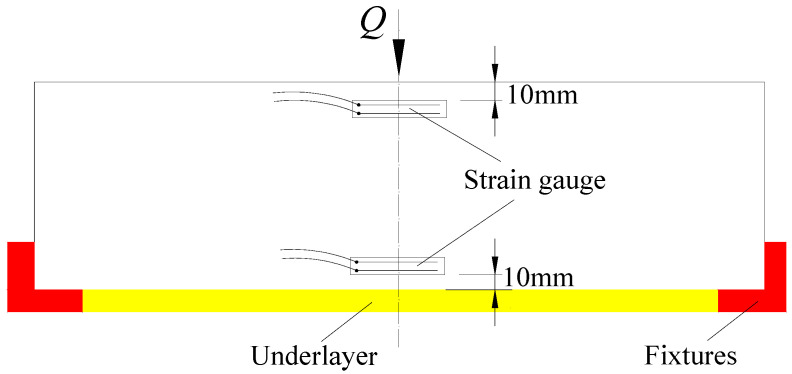
Experimental model diagram.

Due to the unique boundary conditions inherent in tunnel structures, such as low-cycle train loading and the influence of surrounding rock, the behavior of these structures can be significantly affected, making traditional loading devices inadequate for meeting test requirements. To address this, the author undertook specialized research and development to design a loading device tailored to tunnel structures. The device comprises three main components: an axial force component, a formation resistance component, and a lateral restraint component. These components apply axial pressure, simulate formation reactions, and provide horizontal constraints, respectively, effectively simulating the stress conditions of tunnel structure, as illustrated in Figure 1 and Figure 2. Additionally, a resonant load spectrum was applied using an MTS hydraulic servo loading system to simulate subway train loads, with the testing setup depicted in Figure 3. The size of the specimen is 100 mm × 100 mm × 300 mm. Strain gauges (model BQ120-80AA, produced by Zhonghang Electronic Measuring Instruments Co., Ltd., Xi’an, China), each 8 cm in length with a sensitivity of 2.08 and a resistance range of 120 Ω, were firmly adhered to the mid-axis of the specimen’s side surface, positioned 10 mm from both the top and bottom surfaces.

### 2.2. Loading Test Program

(1) Determination of vibration load form and load level

**Figure 4:**
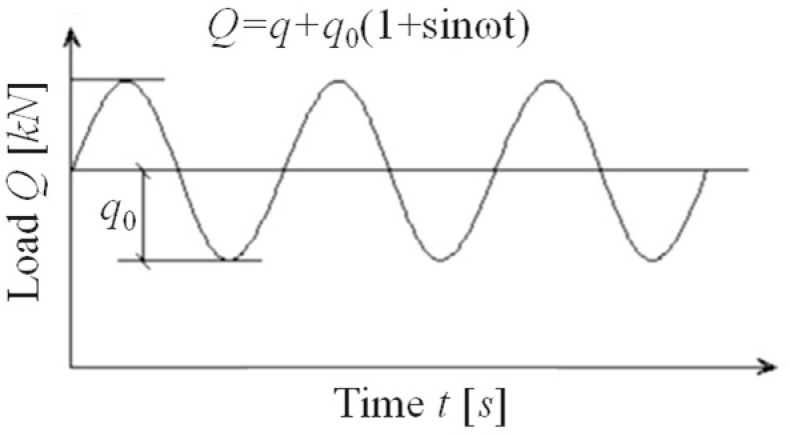
Sinusoidal load curve.

The train load on a tunnel structure is influenced by factors such as the transfer path, transfer time effect, load superposition effect, and track irregularities, resulting in a complex load form. However, based on theoretical foundations like wave theory, the vibration load spectrum of a subway train can be decomposed into a series of resonant loads with varying frequencies and amplitudes. Studying the damage evolution of segmented concrete under these resonant loads can uncover the underlying damage mechanisms caused by subway train loads and enhance our understanding of structural behavior under complex dynamic conditions. Consequently, this test employs a unidirectional constant amplitude sine cyclic loading mode, with the waveform depicted in Figure 4.

**Figure 5:**
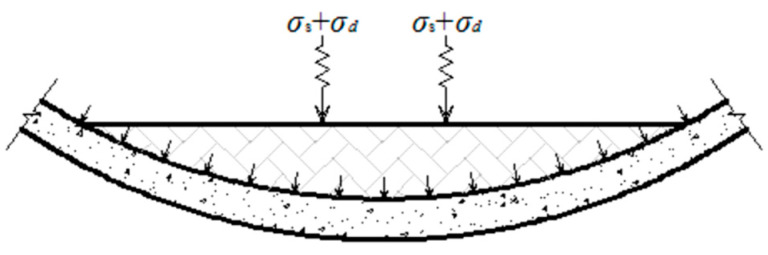
Transmission of train load.

In this test, the train axle load is set at 16 tons, which is the maximum specified in current designs. It is assumed that this axle load is evenly distributed across the tunnel’s bottom structure via the track bed, as illustrated in Figure 5. Following a straightforward conversion, the static load q on the bottom structure is approximately 11.5 kN. Based on the research findings of Ai et al. [34], the maximum dynamic load amplitude of the train load is approximately 20 kN. After simple conversion, the load amplitude on the bottom structure is determined to be about 1.5 kN.

(2) Determination of loading frequency

Train body parameters, operating time, and line conditions all influence train speed and, consequently, the load vibration frequency. Surveys indicate that the design speed of subway trains typically ranges from 80 km/h to 160 km/h. Currently, the maximum operational speed for both the southern section of Shanghai Metro Line 11 and the Hanxi Changlong to Shiqiao section of Guangzhou Metro Line 3 is 120 km/h, marking the highest speed in domestic subway systems. Model A trains are characterized by a single car length of 22.8 m and a bogie center distance of 15.7 m. Table 1 presents the corresponding loading frequencies for various speeds.

Based on the calculations, the train’s loading frequency ranges from 0.3 Hz to 3 Hz. To effectively capture the impact of vibration frequency while maintaining test efficiency, a reference loading frequency of 2 Hz is used in this study.

### 2.3. Prefabrication of Initial Damage Specimens

**Figure 6:**
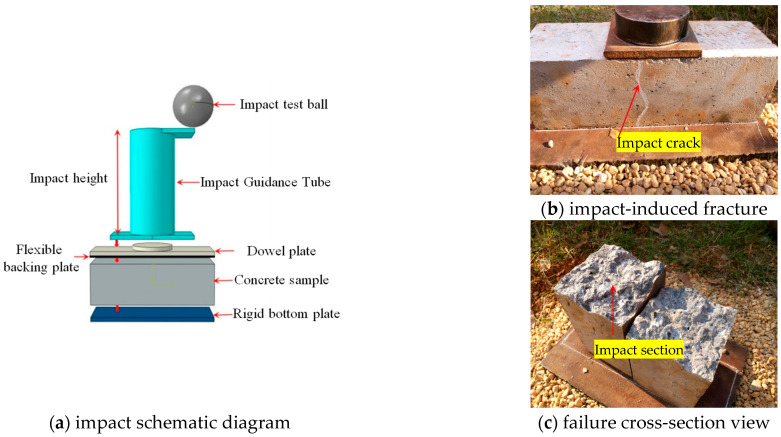
Prefabrication of initial damage in test specimens.

In practical engineering applications, the initial damage to segments primarily arises from impacts during specimen lifting, transportation, and assembly [24,31]. This test replicates such initial damage by employing an impact method, aligning closely with real-world scenarios and enabling the simulation of varying degrees of micro-crack damage within the segments. During the test, a 5 kg impact ball is dropped from a height of 20 cm. To prevent secondary rebound impacts, the specimen base, steel plate, and pebble layer are arranged sequentially, as illustrated in Figure 6. Figure 6b,c illustrate the crack development state and the failure cross-section schematic of the specimen under extreme impact conditions, respectively.

Test results indicate that the concrete specimens can endure approximately 20 impacts. To simulate varying degrees of initial damage, specimens were subjected to 0, 3, 6, 9, and 12 impacts, with a minimum of three specimens per impact level. Additional specimens were tested if data variability was significant.

### 2.4. Loading Scheme and Test Steps

To investigate the fatigue damage behavior of C50 segment concrete with initial damage, five damage levels were established in this study. The test parameters included an axial force of 10 kN, surrounding rock pressure of 2.4 MPa, and a loading frequency of 2 Hz. During multi-stage cyclic loading, the load amplitude remained constant, with each stage increasing by 1 kN from the previous load’s upper and lower limits. The detailed loading scheme is presented in Table 2.

Initially, the dynamic strain gauge was zeroed after applying the segment’s mechanical boundary. A progressive static loading method was then used to gradually increase the load to 13 kN. If significant data dispersion was observed, either the cause was identified or the specimen was replaced. Subsequently, cyclic testing was conducted at each load stage, with each stage comprising 10,000 cycles. The load level was incrementally increased until specimen failure occurred.

## 3. Analysis of Test Results

### 3.1. Test Results and Analysis of Graded Static Loading Section

**Figure 7:**
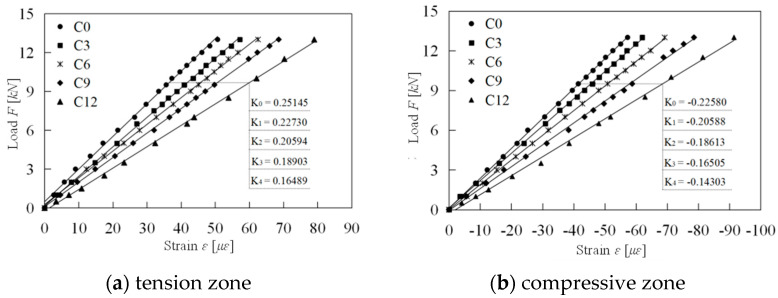
The relationship of load and strain.

Figure 7 depicts the load-strain curves for the segment concrete under static loading, highlighting the tension and compression zones separately.

The primary objective of the graded static load test is to assess the initial damage of the specimen. In this study, the damage variable *D* is defined as follows:

(1) $$D=1-\bar{E}/E_{0}$$

where $\bar{E}$ represents the elastic modulus after damage, and $E_{0}$ denotes the undamaged elastic modulus, referring to the elastic modulus of the non-impacted specimen in this context. The reduction in the slope of the load-strain curve indicates the attenuation of the elastic modulus.

(2) $$D=1-\bar{E}/E_{0}=1-\bar{K}/K_{0}$$

In this context, $\bar{K}$ is the slope of the load-strain curve post-damage, while $K_{0}$ represents the slope of the undamaged load-strain curve.

**Figure 8:**
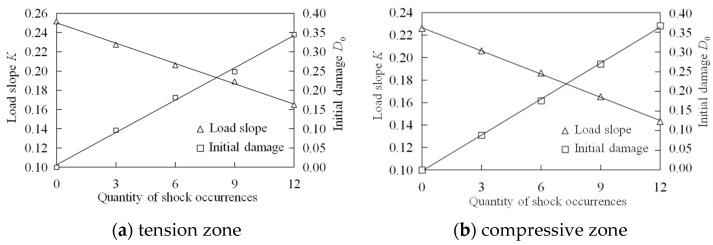
The initial damage of specimen in compressive zone.

Figure 7 illustrates that an increase in the number of impacts on the specimen results in a decreased slope of the load-strain curve, indicating greater initial damage. Figure 8 presents the slope of the load-strain curve and the corresponding initial damage of the specimen at various impact levels. Together, these figures demonstrate a linear relationship between the static load slope or initial damage and the number of impacts on concrete in both the tension and compression zones. Notably, the initial damage values for the tension and compression zones are nearly equal across all impact levels. When the segment concrete is subjected to 12 impacts, both zones reach their maximum damage simultaneously, with values of 0.344 and 0.366, respectively. The similar distribution of initial damage in the tension and compression zones further confirms that the initial damage assessment of prefabricated specimens using the impact method is appropriate.

### 3.2. Test Results and Analysis of Multistage Cyclic Loading Section

(1) Overall analysis

**Figure 9:**
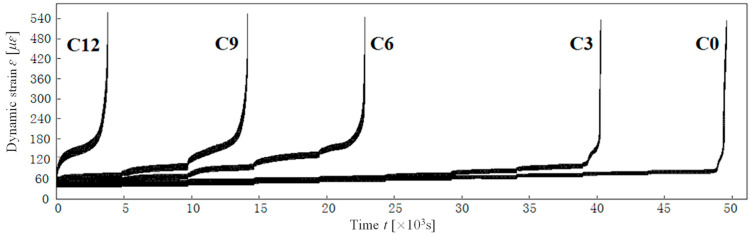
Strain time–history curves of specimens in tension zone under multistage cyclic loading.

**Figure 10:**
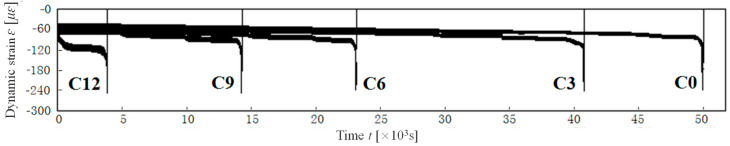
Strain time–history curves of specimens in compressive zone under multistage cyclic loading.

The time–history curves of strain development in both the tension and compression zones of segment concrete under multistage cyclic loading are shown in Figure 9 and Figure 10. The figures reveal a clear consistency and synchronicity in the overall strain development across these zones. However, two key differences are evident.

**Figure 11:**
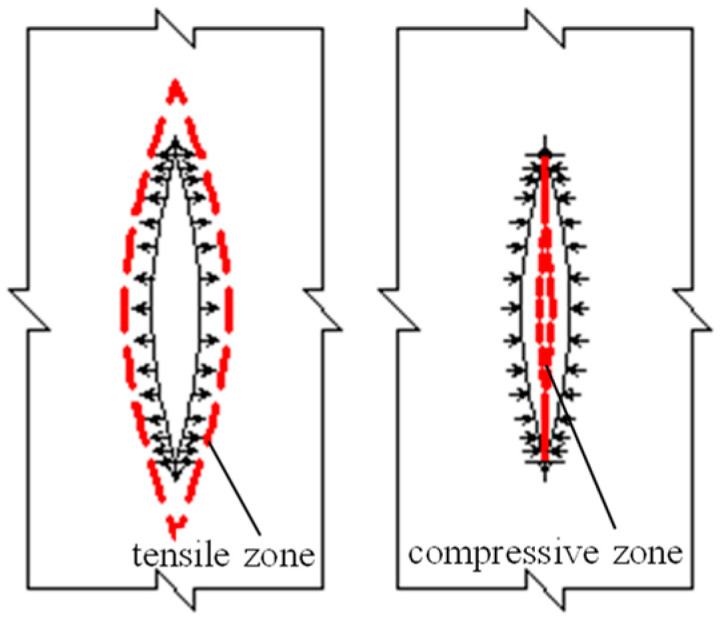
Crack propagation in tension zone and compressive zone.

Firstly, regarding the rate of strain development, the tension zone exhibits a higher rate than the compression zone. This disparity is attributed to the different stress conditions and the inherent tensile and compressive properties of concrete. The mechanism underlying this phenomenon is that micro-defects and micro-cracks in the tensile zone further evolve under tensile stress, leading to a reduction in the effective load-bearing area. Conversely, in the compression zone, micro-defects and micro-cracks tend to close under compressive stress, thereby increasing the effective load-bearing area, as illustrated in Figure 11. This damage mechanism accounts for the variance in strain development between the tension and compression zones, with the difference becoming more pronounced as the damage grade increases.

Secondly, during the rapid failure phase, when the strain in the compression zone reaches approximately 240 με, it swiftly reverses, resulting in failure within 10 to 20 load cycles. Interestingly, even in this rapid failure phase, the compressive stress is only 10% to 20% of the compressive strength, which is insufficient to cause crushing of the concrete. The rapid or even reversed strain development in the compression zone is due to the accelerated propagation of micro-crack clusters in the tension zone. As these cracks extend into the compression zone, the compressive strain transforms into tensile strain, leading to failure within a brief period. Consequently, the damage in both the tension and compression zones is fundamentally tensile in nature.

(2) Average strain

**Figure 12:**
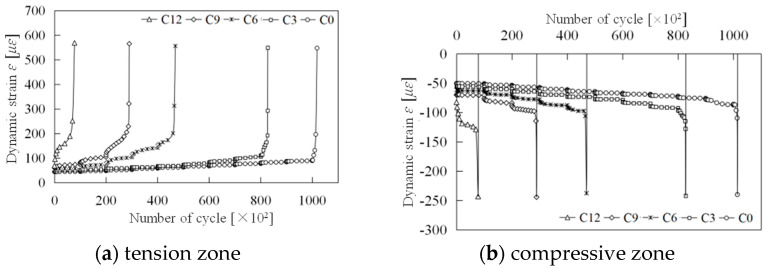
Mean strain of specimens.

**Figure 13:**
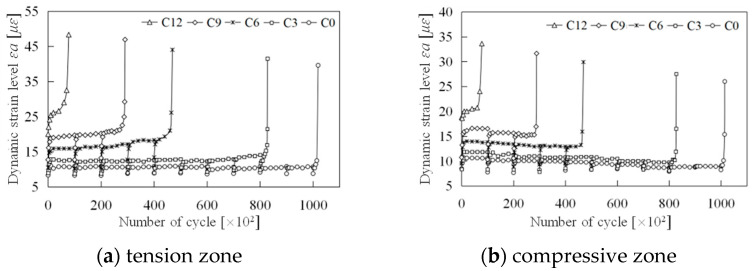
Strain amplitudes of specimens.

To thoroughly investigate the damage mechanisms of segment concrete, it is essential to conduct a deeper analysis of the strain time–history curves presented in Figure 9 and Figure 10. These curves primarily convey two key pieces of information: the magnitude of strain, which can be characterized by the average strain, and the strain amplitude. By converting time into load cycles, the extracted information is illustrated in Figure 12 and Figure 13.

The data from Figure 12 suggest that a higher initial damage grade results in a reduced load threshold at which nonlinear strain development begins in the concrete segment, as well as a decreased maximum load capacity and fewer load cycles it can endure. Additionally, as the load level continues to rise post onset of nonlinear strain development, the nonlinear characteristics become increasingly pronounced. Table 3 provides results of statistical analysis of the load levels corresponding to segments exhibiting significant nonlinear behavior in each group.

The statistical results in Table 3 reveal that the onset of noticeable nonlinear strain development is synchronized between the tension and compression zones. This suggests that the emergence of nonlinearity in the tensile region signifies a marked increase in internal damage, with local micro-defects beginning to interconnect during the nucleation phase. This interconnection contributes to the degradation of the specimen’s macroscopic mechanical properties. As the loading level increases over time, this degradation process accelerates, making the nonlinear characteristics more pronounced. The strain development in the tension zone also impacts the compression zone, aligning with earlier analyses, and thus will not be elaborated upon here.

(3) Strain amplitude

Strain amplitude, as a critical component of the cyclic dynamic strain history curve, plays a significant role in elucidating the damage evolution mechanism of segmented concrete. Changes in average strain further illustrate the intrinsic nature of this damage progression. Figure 13 demonstrates that, at equivalent load levels, specimens with a higher initial damage grade exhibit greater strain amplitudes. Additionally, even for specimens subjected to identical conditions, the strain amplitude evolves subtly as cyclic loading increases. Specifically, there is a gradual increase in the tension zone and a gradual decrease in the compression zone. This behavior indicates that strain amplitude effectively reflects the evolving damage characteristics of the specimens.

Overall, at each load level, the strain amplitude exhibits a distinct three-stage pattern. The first stage, characterized by rapid development and short duration, can be termed the initial development phase. Current explanations for this phase focus on two aspects: the swift evolution of internal micro-defects leading to rapid damage increase, and an absence of new damage, with changes instead attributed to the internal structural adjustment of the material or geometric deformation of existing defects. Based on experimental observations, we propose a more nuanced physical explanation. Before detailing this, it is essential to first examine the second stage, the stable development phase. As seen in Figure 13, once this stage is reached, the strain amplitude in the tension region increases at a consistent micro rate, while in the compression region it behaves inversely. At a constant load level, an increase in strain amplitude signifies a reduction in elastic modulus and an increase in damage, and vice versa. This suggests that during the stable development stage, damage in the tension zone accumulates and progresses, whereas in the compression zone it accumulates and recovers—demonstrating opposing damage evolution processes. This phenomenon has been corroborated by Yang and Xu [35].

Revisiting the initial development stage, it is evident that the strain amplitude experiences a rapid increase at any load level in both the tension and compression zones. This observation indicates that the initial development phase is not characterized by rapid damage development. A plausible explanation is that this stage is a result of a comprehensive reaction. Initially, at each load level, specimens achieve a relatively stable equilibrium through internal structural adjustments and stress redistribution, involving geometric deformation of existing micro-defects without new crack formation or expansion. Additionally, as the target load is applied during testing, the loading equipment gradually increases the generated load, thereby increasing the strain amplitude. This time effect is observable in the test load spectrum and is likely a primary contributor.

The third stage involves rapid damage progression and a swift increase in effective stress, further accelerating the interplay between damage and strain—a view widely accepted in academia [16,17,20].

(4) Damage evolution

**Figure 14:**
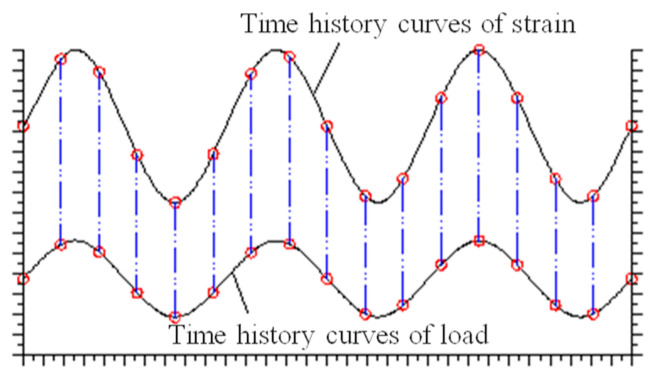
The corresponding relation of strain and load points.

The evolution of concrete damage under cyclic loading has been a longstanding focus of scholarly research. The MTS servo loading system collects data at predetermined time intervals, often leading to challenges in capturing peak and valley values on the cyclic dynamic strain curve, as depicted in Figure 14. When strain amplitude changes are minimal, this significantly impacts the accuracy of data processing. However, every test data point has a corresponding load, ensuring synchronization between the cyclic dynamic strain time–history curve and the cyclic load time–history curve, with consistent initial phase and vibration frequency. Thus, the load associated with each strain data point can be back-calculated to construct the load-strain curve and analyze its slope changes in each cycle.

**Figure 15:**
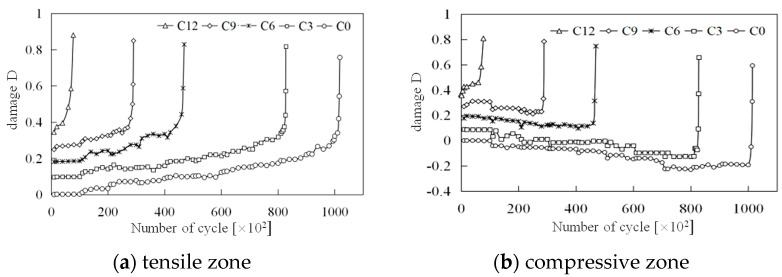
Damage evolution curves.

The damage evolution curves for the tension and compression zones are presented in Figure 15.

Figure 15 provides a clear illustration of the damage evolution patterns in the concrete segments within both the tension and compression zones. In the tension zone, damage consistently increases with the number of load cycles, and this effect is more pronounced at higher load levels. In contrast, in the compression zone, when the initial damage is greater than 0.181 (when the number of impacts is six or more), the damage initially decreases with additional loading cycles but ultimately accelerates failure. Conversely, when the initial damage is less than 0.10 (when the number of impacts is six or fewer), the damage approaches a negative value. According to the previously mentioned definition of non-negative damage, this negative damage does not indicate compressive damage. Instead, it represents a process where cracks in the concrete diminish or even heal under low-cycle compressive loading [35]. However, with subsequent cycles of applied load, failure will also tend to accelerate. As previously noted, the failure of segmented concrete under cyclic loading is primarily due to tensile failure. To further elucidate the mechanism behind the progressive failure due to tensile damage, we conducted a statistical analysis of the average damage evolution rate *dD*/*dN* in the tensile zone for each group of specimens at different load levels, as presented in Table 4.

The data in the table indicate that both load level and initial damage grade significantly affect the average damage evolution rate, *dD*/*dN*. For load levels between 10 and 13 kN, the damage evolution rate for specimens without impact is 7.65 × 10^−8^, whereas for specimens subjected to nine impacts, it rises substantially to 9.16 × 10^−7^, an increase of nearly twelvefold. Moreover, for load levels ranging from 19 to 22 kN, the damage evolution rate based on condition C0 is 70 times greater than that for the 10 to 13 kN range. Consequently, accurately determining the load level and initial damage is crucial in practical fatigue design.

Additionally, due to the quasi-brittle failure characteristics of high-strength concrete, damage progresses swiftly to failure, leaving a remarkably short duration. Therefore, identifying when rapid damage development commences is particularly critical. The transition point between stable and rapid damage evolution phases is defined as the stable damage limit value, *D_k_*, with statistical findings presented in Table 4. The table shows that the stable damage limit *D_k_* generally hovers around 0.4, showing no apparent correlation with the initial damage grade, suggesting that *D_k_* may be a constant value.

Further analysis of the table’s statistical results, while disregarding the number of cycles in the rapid damage development phase, allows for a preliminary estimation of the fatigue life of damaged specimens under various load levels using the calculated value of *D_k_*/(*dD*/*dN*). For instance, at load levels of 10 to 13 kN, the estimated fatigue life for each specimen is approximately: *N_fC_*_0_ ≈ 5.1 million cycles, *N_fC_*_3_ ≈ 2.9 million cycles, *N_fC_*_6_ ≈ 1.44 million cycles, *N_fC_*_9_ ≈ 1.09 million cycles, and *N_fC_*_12_ ≈ 5741 cycles, respectively.

**Figure 16:**
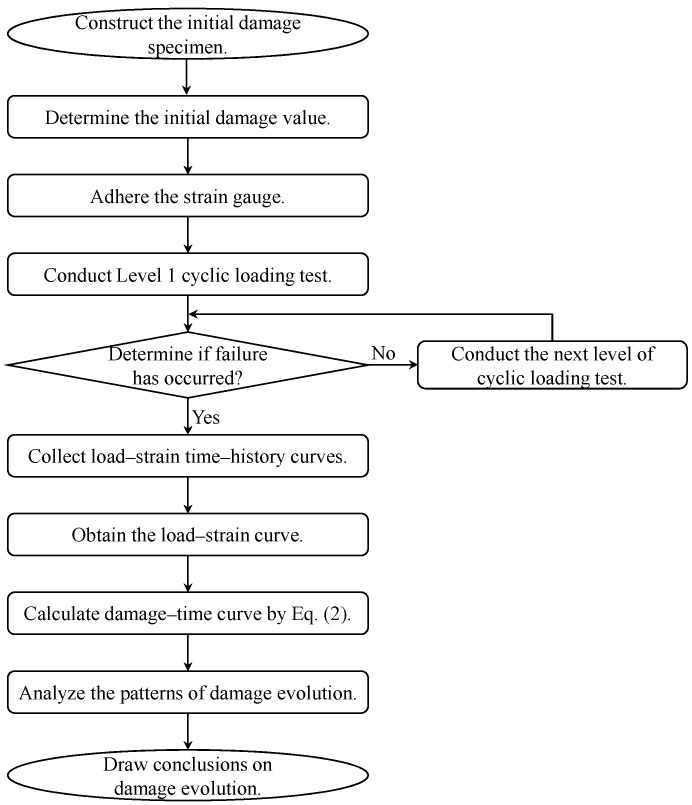
Flowchart of damage analysis for concrete specimens with initial defects under multistage cyclic loading.

Based on the comprehensive analysis presented, the damage analysis process for concrete specimens with initial defects subjected to multistage cyclic loading is illustrated in Figure 16.

**Figure 17:**
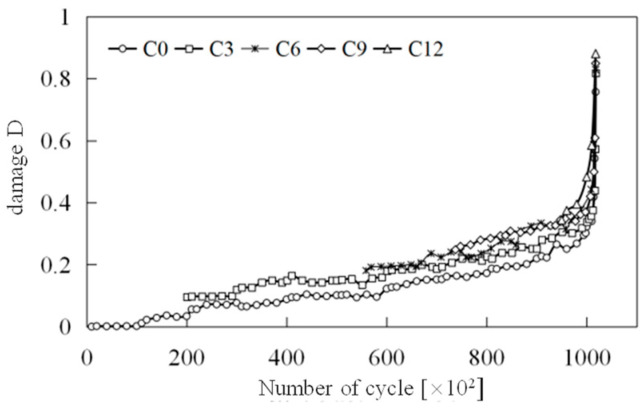
“Memory effect” of damage evolution.

A detailed examination of Figure 15a and alignment of the abscissa of each damage evolution curve at their maximum value (refer to Figure 17) reveals that damage evolution resembles a hysteresis loop with a certain “memory effect”. Notably, as the initial damage grade increases, the damage evolution rate escalates to some extent [7,11]. This implies that using the damage evolution curve of undamaged specimens to predict fatigue life may not be safe, necessitating the inclusion of a safety factor in design. Based on the test results, if the actual initial damage of the segment reaches 0.18, the safety factor should be at least 3.56. Even with an initial damage of only 0.10, a safety factor of 1.77 is recommended.

## 4. Conclusions

(1) As the load level increases, the strain behavior of segmented concrete transitions to a nonlinear pattern. Notably, higher initial damage levels prompt this nonlinear behavior to manifest earlier and reduce the ultimate load capacity. Furthermore, the prominence of the nonlinear phase intensifies with increasing load levels. The loading time in the nonlinear segment accounts for less than 15%, yet it contributes to over 65% of the total damage.
(2) Both the load level and initial damage significantly influence the damage evolution rate, potentially increasing it by an order of magnitude or more. Specifically, for a specimen with an initial damage of 0.096 (C3 condition) subjected to multi-level cyclic loading, the fatigue life decreased by 60% at an initial damage of 0.248 (C9 condition) and by 99% when the initial damage reached 0.344 (C12 condition). Therefore, accurate assessment of load levels and initial segment damage is crucial for reliable fatigue life estimation in the design of shield tunnel structures.
(3) A “stable damage limit” for segment concrete is identified at approximately 0.4, independent of initial damage and load levels. This limit is hypothesized to be a constant. Exceeding this threshold under continuous loading leads to a marked acceleration in the damage evolution rate, culminating in rapid progression towards failure.
(4) The damage evolution in specimens exhibits a “memory effect”, with the initial damage grade influencing the rate of damage progression. Utilizing the damage evolution curve from undamaged specimens for fatigue life predictions is unsafe; incorporating an appropriate safety factor into the design is advised.

This study introduces the novel concept of a stable damage limit and highlights the significant influence of initial damage on nonlinear strain development, offering critical insights for enhancing the fatigue design of structural concrete elements.

## Figures and Tables

**Table 1:** Loading frequency.

Speed [km/h]	Calculated Length [m]	Frequency [Hz]
30	15.7	0.53
	22.8	0.37
80	15.7	1.42
	22.8	0.97
120	15.7	2.12
	22.8	1.46
160	15.7	2.83
	22.8	1.95

**Table 2:** Multistage cyclic loading test programs.

Concrete Sample	Impact Cycle Number	Grouping of Sample	Number of Sample	Cyclic Load	Loading Frequency
Strength: c50 Size: 100 mm × 100 mm × 300 mm	0	C0	≥3	10~13 kN 11~14 kN 12~15 kN …… Loaded 10,000 times each stage until it is destroyed	Loading frequency: 2 Hz Axial force: 10 kN Surrounding rock pressure: 2.4 MPa
	3	C3	≥3		
	6	C6	≥3		
	9	C9	≥3		
	12	C12	≥3		

**Table 3:** Initial Damage Values and Load Levels at which Significant Changes Occur for Each Test Condition.

Grouping of Sample		C0	C3	C6	C9	C12
Compressive zone	Initial damage	0	0.096	0.181	0.248	0.344
	Load [kN]	21~24	15~18	12~15	10~13	10~13
Tensile zone	Initial damage	0	0.088	0.176	0.269	0.366
	Load [kN]	21~24	15~18	12~15	10~13	10~13

**Table 4:** Average damage evolution rate of specimens.

Load Level [kN]	Damage Evolution Rate (*dD*/*dN*) [×10^−6^]				
	C0	C3	C6	C9	C12
10~13	0.08	0.15	0.31	0.92	73.8
11~14	0.81	3.05	4.84	6.18	
12~15	1.75	2.25	5.03	57.9	
13~16	1.77	2.43	7.02		
14~17	1.00	2.02	74.3		
15~18	2.59	3.27			
16~19	2.77	3.73			
17~20	2.16	6.67			
18~21	3.85	173			
19~22	5.47				
20~23	275				
*D_k_*	0.39	0.44	0.44	0.42	0.40

## Data Availability

The original contributions presented in the study are included in the article, further inquiries can be directed to the corresponding author.
